# Supplementary material for: The RNA secondary structure of androgen receptor-FL and V7 transcripts reveals novel regulatory regions
Source: Nucleic Acids Res. 2024 Mar 30;52(11):6596–613. doi: 10.1093/nar/gkae220 (PMC11194067; doi:10.1093/nar/gkae220)

AR_M12_-1_5U_1

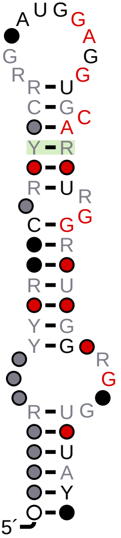

Supplement: gkae220_Supplemental_Files [file gkae220_supplemental_files.zip › Supplementary File S5.zip!/Supplementary File S5/FL_RScape.zip!/FL_RScape/AR_M12_-1_5U_1.R2R.sto.pdf]
